# Supplementary material for: Responses to simulated nitrogen deposition by the neotropical epiphytic orchid Laelia speciosa
Source: PeerJ. 2015 Jun 23;3:e1021. doi: 10.7717/peerj.1021 (PMC4485242; doi:10.7717/peerj.1021)
Supplement: Supplemental Information 1 [file peerj-03-1021-s001.doc]

**Flowers**

Kruskal-Wallis non-parametric Analysis of Variance

Normality Test: Failed (P < 0.050)

Equal Variance Test: Passed (P = 0.574)

Group Name N Missing Mean Std Dev SEM

T1 21 0 0.476 1.123 0.245

T2 21 0 0.571 1.326 0.289

T3 21 0 0.810 1.721 0.376

T4 21 0 1.476 2.909 0.635

T5 21 0 0.381 0.921 0.201

T5 21 0 0.381 0.921 0.201

T6 21 0 0.000 0.000 0.000

Source of Variation DF SS MS F P

Between Groups 5 26.925 4.488 1.946 0.077

Residual 140 322.762 2.305

Total 146 349.687

The differences in the mean values among the treatment groups are not great enough to exclude the possibility that the difference is due to random sampling variability; there is not a statistically significant difference (P = 0.077).

Power of performed test with alpha = 0.050: 0.359

The power of the performed test (0.359) is below the desired power of 0.800.

Less than desired power indicates you are less likely to detect a difference when one actually exists.

Negative results should be interpreted cautiously.

**Pseudobulbs**

Kruskal-Wallis non-parametric One Way Analysis of Variance

Normality Test: Failed (P < 0.050)

Equal Variance Test: Passed (P = 0.058)

Group Name N Missing Mean Std Dev SEM

T1 20 0 0.900 0.308 0.0688

T2 20 0 0.950 0.224 0.0500

T3 20 0 1.000 0.324 0.0725

T4 20 0 1.300 0.470 0.105

T5 20 0 0.700 0.470 0.105

T6 20 0 0.650 0.489 0.109

Source of Variation DF SS MS F P

Between Groups 5 5.467 1.093 7.042 <0.001

Residual 114 17.700 0.155

Total 119 23.167

All Pairwise Multiple Comparison Procedures (Tukey method):

Overall significance level = 0.05

Comparisons for factor:

Comparison Diff of Means t Unadjusted P Critical Level Significant?

T4 vs. T6 0.650 5.216 0.000000825 0.003 Yes

T4 vs. T5 0.600 4.815 0.00000456 0.004 Yes

T4 vs. T1 0.400 3.210 0.00172 0.004 Yes

T3 vs. T6 0.350 2.809 0.00585 0.004 Yes

T4 vs. T2 0.350 2.809 0.00585 0.005 Yes

T4 vs. T3 0.300 2.408 0.0177 0.005 Yes

T3 vs. T5 0.300 2.408 0.0177 0.006 No

T2 vs. T6 0.300 2.408 0.0177 0.006 No

T1 vs. T6 0.250 2.006 0.0472 0.007 No

T2 vs. T5 0.250 2.006 0.0472 0.009 No

T1 vs. T5 0.200 1.605 0.111 0.010 No

T3 vs. T1 0.1000 0.803 0.424 0.013 No

T2 vs. T1 0.0500 0.401 0.689 0.017 No

T3 vs. T2 0.0500 0.401 0.689 0.025 No

T5 vs. T6 0.0500 0.401 0.689 0.050 No

**Leaves**

Kruskal-Wallis non-parametric One Way Analysis of Variance

Normality Test: Failed (P < 0.050)

Equal Variance Test: Passed (P < 0.057)

Group Name N Missing Mean Std Dev SEM

T1 20 0 0.900 0.308 0.0688

T2 20 0 1.050 0.224 0.0500

T3 20 0 1.100 0.308 0.0688

T4 20 0 1.350 0.489 0.109

T5 20 0 0.750 0.444 0.0993

T6 20 0 0.650 0.489 0.109

Source of Variation DF SS MS F P

Between Groups 5 6.467 1.293 8.474 <0.001

Residual 114 17.400 0.153

Total 119 23.867

The differences in the mean values among the treatment groups are greater than would be expected by chance; there is a statistically significant difference (P = <0.001).

Power of performed test with alpha = 0.050: 1.000

All Pairwise Multiple Comparison Procedures (Tukey method):

Overall significance level = 0.05

Comparisons for factor:

Comparison Diff of Means t Unadjusted P Critical Level Significant?

T4 vs. T6 0.700 5.666 0.000000111 0.003 Yes

T4 vs. T5 0.600 4.857 0.00000383 0.004 Yes

T4 vs. T1 0.450 3.642 0.000408 0.004 Yes

T3 vs. T6 0.450 3.642 0.000408 0.004 Yes

T2 vs. T6 0.400 3.238 0.00158 0.005 Yes

T3 vs. T5 0.350 2.833 0.00545 0.005 Yes

T4 vs. T2 0.300 2.428 0.0167 0.006 No

T2 vs. T5 0.300 2.428 0.0167 0.006 No

T1 vs. T6 0.250 2.024 0.0454 0.007 No

T4 vs. T3 0.250 2.024 0.0454 0.009 No

T3 vs. T1 0.200 1.619 0.108 0.010 No

T2 vs. T1 0.150 1.214 0.227 0.013 No

T1 vs. T5 0.150 1.214 0.227 0.017 No

T5 vs. T6 0.1000 0.809 0.420 0.025 No

T3 vs. T2 0.0500 0.405 0.686 0.050 No

**Fv/Fm**

One Way Analysis of Variance

Normality Test: Passed (P = 0.185)

Equal Variance Test: Failed (P < 0.050)

Group Name N Missing Mean Std Dev SEM

T1 5 0 0.770 0.0134 0.00598

T2 5 0 0.799 0.00687 0.00307

T3 5 0 0.828 0.0440 0.0197

T4 5 0 0.854 0.0179 0.00801

T5 5 0 0.657 0.0253 0.0113

T6 5 0 0.605 0.0218 0.00973

Source of Variation DF SS MS F P

Between Groups 5 0.247 0.0495 82.571 <0.001

Residual 24 0.0144 0.000599

Total 29 0.262

The differences in the mean values among the treatment groups are greater than would be expected by chance; there is a statistically significant difference (P = <0.001).

Power of performed test with alpha = 0.050: 1.000

All Pairwise Multiple Comparison Procedures (Holm-Sidak method):

Overall significance level = 0.05

Comparisons for factor:

Comparison Diff of Means t Unadjusted P Critical Level Significant?

T4 vs. T6 0.250 16.122 2.233E-014 0.003 Yes

T3 vs. T6 0.223 14.427 2.528E-013 0.004 Yes

T4 vs. T5 0.197 12.722 3.696E-012 0.004 Yes

T2 vs. T6 0.195 12.570 4.755E-012 0.004 Yes

T3 vs. T5 0.171 11.026 7.059E-011 0.005 Yes

T1 vs. T6 0.166 10.697 0.000000000130 0.005 Yes

T2 vs. T5 0.142 9.169 0.00000000260 0.006 Yes

T1 vs. T5 0.113 7.296 0.000000155 0.006 Yes

T4 vs. T1 0.0840 5.426 0.0000142 0.007 No

T3 vs. T1 0.0578 3.730 0.00104 0.009 No

T4 vs. T2 0.0550 3.553 0.00162 0.010 No

T5 vs. T6 0.0526 3.401 0.00235 0.013 No

T2 vs. T1 0.0290 1.873 0.0733 0.017 No

T3 vs. T2 0.0288 1.857 0.0756 0.025 No

T4 vs. T3 0.0262 1.696 0.103 0.050 No

**Total Chlorophyll**

One Way Analysis of Variance

Normality Test: Passed (P = 0.246)

Equal Variance Test: Passed (P < 0.053)

Group Name N Missing Mean Std Dev SEM

T1 4 0 0.456 0.0277 0.0139

T2 4 0 0.462 0.0813 0.0406

T3 4 0 0.543 0.0330 0.0165

T4 4 0 0.664 0.0702 0.0351

T5 4 0 0.432 0.00710 0.00355

T6 4 0 0.415 0.00756 0.00378

Source of Variation DF SS MS F P

Between Groups 5 0.176 0.0353 15.683 <0.001

Residual 18 0.0405 0.00225

Total 23 0.217

The differences in the mean values among the treatment groups are greater than would be expected by chance; there is a statistically significant difference (P = <0.001).

Power of performed test with alpha = 0.050: 1.000

All Pairwise Multiple Comparison Procedures (Holm-Sidak method):

Overall significance level = 0.05

Comparisons for factor:

Comparison Diff of Means t Unadjusted P Critical Level Significant?

T4 vs. T6 0.250 7.450 0.000000666 0.003 Yes

T4 vs. T5 0.233 6.941 0.00000174 0.004 Yes

T4 vs. T1 0.209 6.224 0.00000715 0.004 Yes

T4 vs. T2 0.203 6.041 0.0000104 0.004 Yes

T3 vs. T6 0.128 3.818 0.00126 0.005 Yes

T4 vs. T3 0.122 3.632 0.00191 0.005 Yes

T3 vs. T5 0.111 3.308 0.00391 0.006 Yes

T3 vs. T1 0.0869 2.591 0.0084 0.006 Yes

T3 vs. T2 0.0808 2.409 0.0269 0.007 No

T2 vs. T6 0.0472 1.409 0.176 0.009 No

T1 vs. T6 0.0411 1.226 0.236 0.010 No

T2 vs. T5 0.0302 0.899 0.380 0.013 No

T1 vs. T5 0.0240 0.717 0.483 0.017 No

T5 vs. T6 0.0171 0.510 0.617 0.025 No

T2 vs. T1 0.00611 0.182 0.857 0.050 No

**Chla**

One Way Analysis of Variance

Normality Test: Passed (P = 0.594)

Equal Variance Test: Passed (P = 0.585)

Group Name N Missing Mean Std Dev SEM

T1 4 0 0.111 0.0262 0.0131

T2 4 0 0.117 0.0225 0.0112

T3 4 0 0.154 0.0361 0.0181

T4 4 0 0.176 0.0223 0.0111

T5 4 0 0.114 0.0131 0.00655

T6 4 0 0.0986 0.00516 0.00258

Source of Variation DF SS MS F P

Between Groups 5 0.0177 0.00354 6.667 0.001

Residual 18 0.00957 0.000532

Total 23 0.0273

The differences in the mean values among the treatment groups are greater than would be expected by chance; there is a statistically significant difference (P = 0.001).

Power of performed test with alpha = 0.050: 0.965

All Pairwise Multiple Comparison Procedures (Holm-Sidak method):

Overall significance level = 0.05

Comparisons for factor:

Comparison Diff of Means t Unadjusted P Critical Level Significant?

T4 vs. T6 0.0774 4.745 0.000162 0.003 Yes

T4 vs. T1 0.0653 4.003 0.000834 0.004 Yes

T4 vs. T5 0.0619 3.797 0.00132 0.004 Yes

T4 vs. T2 0.0590 3.618 0.00197 0.004 Yes

T3 vs. T6 0.0549 3.368 0.01342 0.005 NO

T3 vs. T1 0.0428 2.626 0.0171 0.005 No

T3 vs. T5 0.0395 2.420 0.0263 0.006 No

T3 vs. T2 0.0365 2.241 0.0379 0.006 No

T4 vs. T3 0.0225 1.377 0.185 0.007 No

T2 vs. T6 0.0184 1.127 0.274 0.009 No

T5 vs. T6 0.0155 0.948 0.355 0.010 No

T1 vs. T6 0.0121 0.742 0.468 0.013 No

T2 vs. T1 0.00628 0.385 0.705 0.017 No

T5 vs. T1 0.00336 0.206 0.839 0.025 No

T2 vs. T5 0.00291 0.179 0.860 0.050 No

**Chlb**

One Way Analysis of Variance

Normality Test: Passed (P = 0.594)

Equal Variance Test: Failed (P < 0.050)

Group Name N Missing Mean Std Dev SEM

T1 4 0 0.345 0.00455 0.00228

T2 4 0 0.345 0.0589 0.0295

T3 4 0 0.389 0.0261 0.0131

T4 4 0 0.489 0.0736 0.0368

T5 4 0 0.318 0.0130 0.00652

T6 4 0 0.316 0.00324 0.00162

Source of Variation DF SS MS F P

Between Groups 5 0.0851 0.0170 10.447 <0.001

Residual 18 0.0293 0.00163

Total 23 0.114

The differences in the mean values among the treatment groups are greater than would be expected by chance; there is a statistically significant difference (P = <0.001).

Power of performed test with alpha = 0.050: 0.999

All Pairwise Multiple Comparison Procedures (Holm-Sidak method):

Overall significance level = 0.05

Comparisons for factor:

Comparison Diff of Means t Unadjusted P Critical Level Significant?

T4 vs. T6 0.173 6.046 0.0000103 0.003 Yes

T4 vs. T5 0.171 5.989 0.0000115 0.004 Yes

T4 vs. T2 0.144 5.034 0.0000863 0.004 Yes

T4 vs. T1 0.143 5.028 0.0000874 0.004 Yes

T4 vs. T3 0.0994 3.482 0.00266 0.005 Yes

T3 vs. T6 0.0732 2.563 0.0195 0.005 No

T3 vs. T5 0.0715 2.506 0.0220 0.006 No

T3 vs. T2 0.0443 1.551 0.138 0.006 No

T3 vs. T1 0.0441 1.546 0.140 0.007 No

T1 vs. T6 0.0290 1.018 0.322 0.009 No

T2 vs. T6 0.0289 1.012 0.325 0.010 No

T1 vs. T5 0.0274 0.960 0.350 0.013 No

T2 vs. T5 0.0272 0.955 0.352 0.017 No

T5 vs. T6 0.00163 0.0572 0.955 0.025 No

T1 vs. T2 0.000163 0.00570 0.996 0.050 No

**δ^13^C**

One Way Analysis of Variance

Normality Test: Passed (P = 0.781)

Equal Variance Test: Passed (P = 0.658)

Group Name N Missing Mean Std Dev SEM

T1 4 0 14.506 0.153 0.0764

T2 4 0 14.623 0.147 0.0734

T3 4 0 14.460 0.0599 0.0299

T4 4 0 14.650 0.138 0.0689

T5 4 0 14.377 0.142 0.0712

T6 4 0 14.449 0.118 0.0588

Source of Variation DF SS MS F P

Between Groups 5 0.225 0.0449 2.656 0.057

Residual 18 0.305 0.0169

Total 23 0.529

The differences in the mean values among the treatment groups are not great enough to exclude the possibility that the difference is due to random sampling variability; there is not a statistically significant difference (P = 0.057).

Power of performed test with alpha = 0.050: 0.442

The power of the performed test (0.442) is below the desired power of 0.800.

Less than desired power indicates you are less likely to detect a difference when one actually exists. Negative results should be interpreted cautiously.

**%C**

One Way Analysis of Variance

Data source: Data 1 in Notebook 1

Normality Test: Passed (P = 0.476)

Equal Variance Test: Passed (P = 0.874)

Group Name N Missing Mean Std Dev SEM

T1 4 0 44.828 0.705 0.352

T2 4 0 44.417 0.231 0.116

T3 4 0 45.114 0.413 0.207

T4 4 0 46.200 0.509 0.255

T5 4 0 46.025 0.685 0.343

T6 4 0 45.168 0.574 0.287

Source of Variation DF SS MS F P

Between Groups 5 9.558 1.912 6.445 0.001

Residual 18 5.339 0.297

Total 23 14.897

The differences in the mean values among the treatment groups are greater than would be expected by chance; there is a statistically significant difference (P = 0.001).

Power of performed test with alpha = 0.050: 0.957

All Pairwise Multiple Comparison Procedures (Holm-Sidak method):

Overall significance level = 0.05

Comparisons for factor:

Comparison Diff of Means t Unadjusted P Critical Level Significant?

T4 vs. T2 1.783 4.630 0.000208 0.003 Yes

T5 vs. T2 1.608 4.176 0.000568 0.004 Yes

T4 vs. T1 1.372 3.562 0.00223 0.004 Yes

T5 vs. T1 1.197 3.108 0.00507 0.004 Yes

T4 vs. T3 1.086 2.819 0.0064 0.005 Yes

T4 vs. T6 1.032 2.679 0.0153 0.005 No

T5 vs. T3 0.911 2.365 0.0295 0.006 No

T5 vs. T6 0.857 2.225 0.0391 0.006 No

T6 vs. T2 0.751 1.951 0.0668 0.007 No

T3 vs. T2 0.697 1.811 0.0869 0.009 No

T1 vs. T2 0.411 1.068 0.300 0.010 No

T6 vs. T1 0.340 0.883 0.389 0.013 No

T3 vs. T1 0.286 0.743 0.467 0.017 No

T4 vs. T5 0.175 0.454 0.655 0.025 No

T6 vs. T3 0.0540 0.140 0.890 0.050 No

**%N**

One Way Analysis of Variance

Normality Test: Passed (P = 0.305)

Equal Variance Test: Passed (P = 0.980)

Group Name N Missing Mean Std Dev SEM

T1 4 0 1.178 0.0651 0.0325

T2 4 0 1.220 0.0987 0.0494

T3 4 0 1.279 0.0600 0.0300

T4 4 0 1.353 0.0541 0.0270

T5 4 0 1.745 0.0509 0.0254

T6 4 0 2.391 0.0818 0.0409

Source of Variation DF SS MS F P

Between Groups 5 4.407 0.881 177.514 <0.001

Residual 18 0.0894 0.00497

Total 23 4.496

The differences in the mean values among the treatment groups are greater than would be expected by chance; there is a statistically significant difference (P = <0.001).

Power of performed test with alpha = 0.050: 1.000

All Pairwise Multiple Comparison Procedures (Holm-Sidak method):

Overall significance level = 0.05

Comparisons for factor:

Comparison Diff of Means t Unadjusted P Critical Level Significant?

T6 vs. T1 1.213 24.348 3.150E-015 0.003 Yes

T6 vs. T2 1.171 23.505 5.831E-015 0.004 Yes

T6 vs. T3 1.112 22.311 1.447E-014 0.004 Yes

T6 vs. T4 1.038 20.824 4.794E-014 0.004 Yes

T6 vs. T5 0.646 12.966 0.000000000144 0.005 Yes

T5 vs. T1 0.567 11.383 0.00000000118 0.005 Yes

T5 vs. T2 0.525 10.540 0.00000000395 0.006 Yes

T5 vs. T3 0.466 9.346 0.0000000250 0.006 Yes

T5 vs. T4 0.392 7.858 0.000000316 0.007 Yes

T4 vs. T1 0.176 3.525 0.00242 0.009 Yes

T4 vs. T2 0.134 2.682 0.0152 0.010 No

T3 vs. T1 0.101 2.037 0.0567 0.013 No

T4 vs. T3 0.0741 1.488 0.154 0.017 No

T3 vs. T2 0.0595 1.194 0.248 0.025 No

T2 vs. T1 0.0420 0.843 0.410 0.050 No

**δ^15^N**

One Way Analysis of Variance

Normality Test: Passed (P = 0.533)

Equal Variance Test: Passed (P = 0.178)

Group Name N Missing Mean Std Dev SEM

T1 4 0 2.783 0.152 0.0760

T2 4 0 3.011 0.340 0.170

T4 4 0 3.732 0.303 0.151

T5 4 0 3.857 0.457 0.229

T6 4 0 4.243 0.366 0.183

Source of Variation DF SS MS F P

Between Groups 5 5.934 1.484 15.68 <0.001

Residual 15 1.720 0.115

Total 19 7.654

The differences in the mean values among the treatment groups are greater than would be expected by chance;

there is a statistically significant difference (P = <0.001).

Power of performed test with alpha = 0.050: 0.999

All Pairwise Multiple Comparison Procedures (Holm-Sidak method):

Overall significance level = 0.05

Comparisons for factor:

Comparison Diff of Means t Unadjusted P Critical Level Significant?

T6 vs. T1 1.460 6.099 0.0000204 0.005 Yes

T6 vs. T2 1.232 5.148 0.000119 0.005 Yes

T6 vs. T3 0.511 5.140 0.000261 0.005 Yes

T6 vs. T4 0.655 5.135 0.00049 0.005 Yes

T6 vs. T5 0.387 4.964 0.00045 0.003 yes

T5 vs. T1 1.073 4.483 0.00043 0.006 Yes

T4 vs. T1 0.949 3.964 0.00125 0.006 Yes

T5 vs. T3 0.089 3.667 0.00115 0.006 Yes

T5 vs. T2 0.846 3.532 0.00302 0.009 Yes

T4 vs. T2 0.721 3.013 0.00875 0.010 Yes

T5 vs. T4 0.124 2.519 0.00611 0.050 Yes

T4 vs. T3 0.387 1.616 0.127 0.017 No

T3 vs. T1 0.315 1.373 0.243 0.019 No

T3 vs. T2 0.235 1.014 0.254 0.023 No

T2 vs. T1 0.228 0.951 0.357 0.025 No
